# MetaLo: metabolic analysis of Logical models extracted from molecular interaction maps

**DOI:** 10.1515/jib-2023-0048

**Published:** 2024-02-06

**Authors:** Sahar Aghakhani, Anna Niarakis, Sylvain Soliman

**Affiliations:** GenHotel – European Research Laboratory for Rheumatoid Arthritis, Univ. Evry, Univ. Paris-Saclay, Evry, France; Lifeware Group, Inria Saclay, Palaiseau, France

**Keywords:** Boolean model, computational biology, hybrid modeling, metabolism, molecular interaction map

## Abstract

Molecular interaction maps (MIMs) are static graphical representations depicting complex biochemical networks that can be formalized using one of the Systems Biology Graphical Notation languages. Regardless of their extensive coverage of various biological processes, they are limited in terms of dynamic insights. However, MIMs can serve as templates for developing dynamic computational models. We present MetaLo, an open-source Python package that enables the coupling of Boolean models inferred from process description MIMs with generic core metabolic networks. MetaLo provides a framework to study the impact of signaling cascades, gene regulation processes, and metabolic flux distribution of central energy production pathways. MetaLo computes the Boolean model’s asynchronous asymptotic behavior, through the identification of trap-spaces, and extracts metabolic constraints to contextualize the generic metabolic network. MetaLo is able to handle large-scale Boolean models and genome-scale metabolic models without requiring kinetic information or manual tuning. The framework behind MetaLo enables in depth analysis of the regulatory model, and may allow tackling a lack of omics data in poorly addressed biological fields to contextualize generic metabolic networks along with improper automatic reconstructions of cell- and/or disease-specific metabolic networks. MetaLo is available at https://pypi.org/project/metalo/ under the terms of the GNU General Public License v3.

## 1 Introduction

Molecular interaction maps (MIMs) [1] are static graphical representations depicting complex biochemical networks that can be formalized using one of the Systems Biology Graphical Notation languages. Regardless of their extensive coverage of various biological processes, they are limited in terms of dynamic insights. However, MIMs can serve as templates for developing dynamic computational models [2–7]. We present MetaLo, an open-source Python package that enables the coupling of Boolean models inferred from process description MIMs with generic core metabolic networks. MetaLo provides a framework to study the impact of signaling cascades, gene regulation processes, and metabolic flux distribution of central energy production pathways. MetaLo computes the Boolean model’s asynchronous asymptotic behavior, through the identification of trap-spaces, and extracts metabolic constraints to contextualize the generic metabolic network. MetaLo is able to handle large-scale Boolean models and genome-scale metabolic models without requiring kinetic information or manual tuning. The framework behind MetaLo enables in depth analysis of the regulatory model, and may allow tackling a lack of omics data in poorly addressed biological fields to contextualize generic metabolic networks along with improper automatic reconstructions of cell- and/or disease-specific metabolic networks. MetaLo is available at https://pypi.org/project/metalo/ under the terms of the GNU General Public License v3.

## 2 Methods

**Figure 1:**
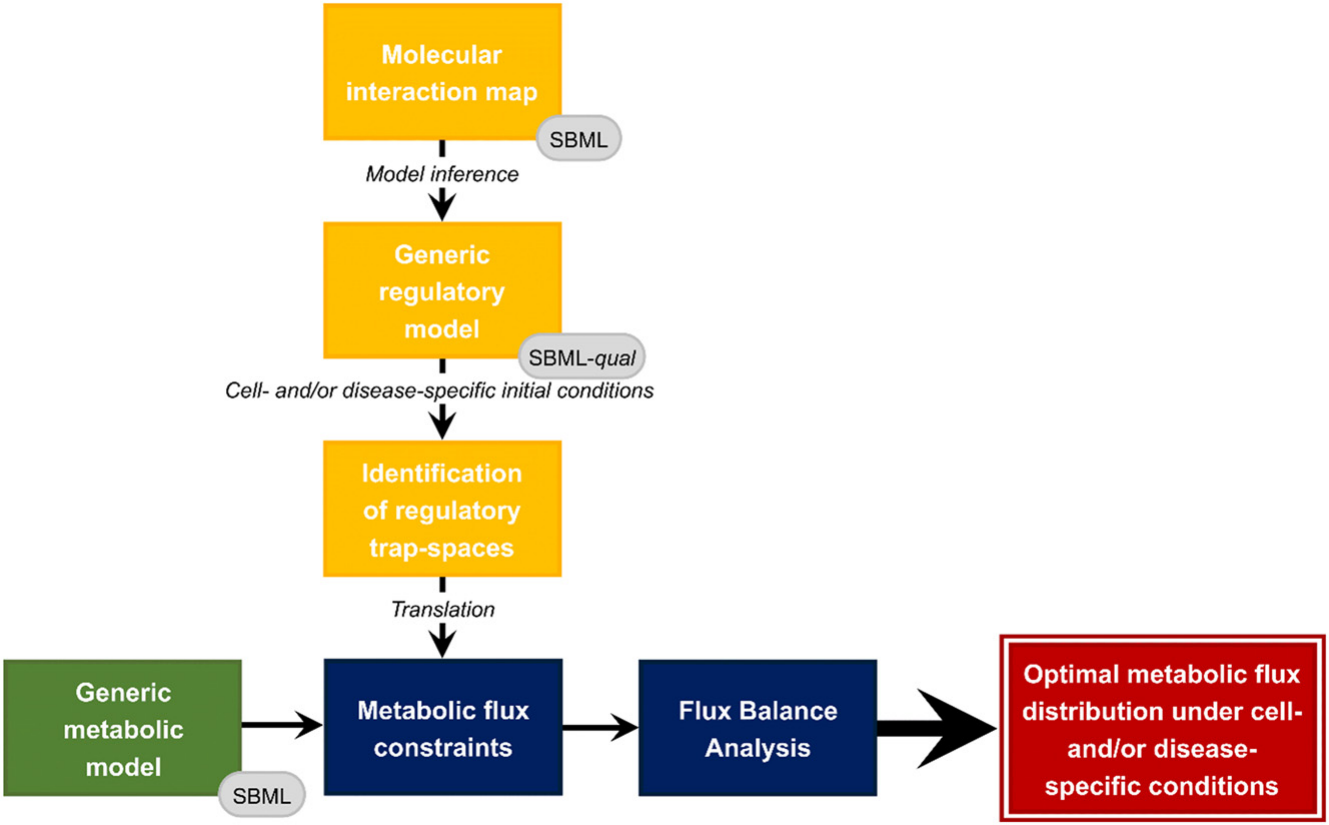
General architecture of MetaLo, adapted from [8].

MetaLo’s general architecture is thoroughly detailed in [8] and presented in Figure 1. It allows the integration of a cell- and disease-specific regulatory model with a generic reconstruction of human central metabolism in a context-specific manner through extraction of constraints from metabolic components with a proven inactive state. In greater detail, the MIM is used as a basis to infer a regulatory Boolean model by CaSQ’s map-to-model framework [9]. The regulatory model’s trap-spaces are computed through Trappist [10], reflecting the model’s asymptotic behavior, and particularly the asymptotic behavior of metabolic components (i.e. metabolic enzymes and metabolites) under regulatory conditions. The value of trap-spaces relative to metabolic components are used to constrain the associated metabolic network’s fluxes in the generic metabolic model. For every metabolic component with a projected maximal regulatory trap-space value equal to 0, the flux of its associated metabolic reactions (i.e. catalyzed reactions for enzymes or producing reactions for metabolites) are constrained to 0. Two FBAs are performed using CobraPy [11] to evaluate the generic metabolic model’s flux distribution in a control state (without any additional metabolic constraints) and a cell- and/or disease-specific state (with additional metabolic constraints). Both FBAs are set with an objective function of maximum cellular ATP production to reflect the primary energy production role of metabolism. FBA results are interpreted in terms of flux distribution through the ratios of ATP production from glycolysis or oxidative pathways relative to total ATP production to highlight the main metabolic pathway of ATP production in each situation.

## 3 Implementation

Written in Python, MetaLo is a pip-installable package intended to couple Boolean models inferred from process description MIMs with generic metabolic networks. It allows assessing the impact of extracellular stimuli on cellular signaling, gene regulation processes, and metabolic flux distribution, particularly its central function of energy production in cell- and/or disease-specific contexts.

The packaging of the initial framework responds to the necessity of expanding its applicability beyond the original studies it was conceived for and to a broader spectrum of systems biology studies. This strategic evolution, making the framework automatic and introducing a graphical interface, positions MetaLo as a flexible and powerful tool for researchers seeking to delve into different layers of biological systems and their intricate interactions. The automation facilitated by the packaging enhances MetaLo’s accessibility, making it particularly valuable for a wide audience that may not have in-depth knowledge of Python. MetaLo’s significance is accentuated in a context where MIMs are increasingly employed, and there is a growing demand for more comprehensive dynamic studies.

While we use the same global approach in the initial framework and MetaLo, the latter presents some differences in terms of the tools and libraries used to improve performance and facilitate packaging and distribution. For instance, the previous step of application of CoLoMoTo’s [12] value propagation algorithm [13], to reduce the complexity of the regulatory model before identifying its trap-spaces through the Java bioLQM toolkit [14], is no longer necessary. The search for trap-spaces is now handled by Trappist [10], an open-source Python tool for computing Boolean model’s minimal trap-spaces that, additionally, achieves the computation of trap-spaces faster.

**Figure 2:**
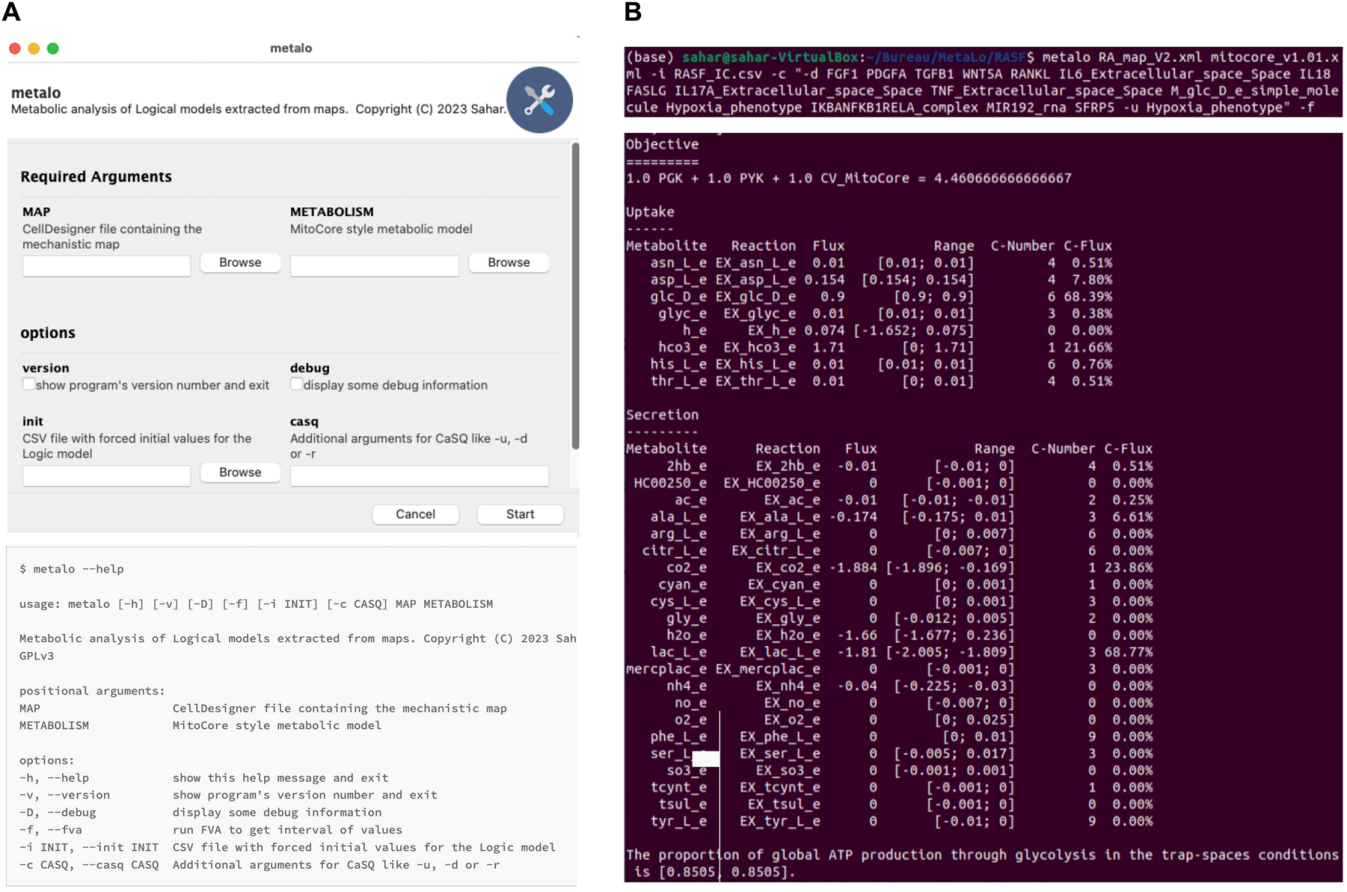
MetaLo graphical interface. (A) Usage of MetaLo through the graphical interface or the command line. Minimal inputs required are a cell- and/or disease-specific molecular interaction map in the CellDesigner XML file format and a generic constraint-based metabolic network in the SBML format. Additional arguments may be specified including initial conditions for the simulation of the regulatory model, specification for model inference through CaSQ or conduction of flux variability analysis instead of flux balance analysis. (B) MetaLo analysis of RASFs regulatory impact upon metabolic processes. On top is the specification of analysis parameters with the generic RA-map V2 and MitoCore’s metabolic model, RASF-specific initial conditions, CaSQ arguments for inference of the RASF model, and running flux variability analysis instead of flux balance analysis. Below are displayed flux variability analysis results for main uptake and secretion metabolic reaction fluxes and in terms of overall ratio of ATP produced through glycolytic pathways.

MetaLo is published under the GNU General Public License version 3.0 (https://pypi.org/project/metalo/). A multiplatform graphical user interface is implemented using the wxPython toolkit (https://wxpython.org/) and the Gooey parser (https://pypi.org/project/Gooey/). Thus, users do not need to be proficient in the Python programming language as MetaLo can be operated in two ways, via the command line or its graphical interface (Figure 2).

MetaLo is interoperable with a wide range of systems biology tools as the input and output files required are in standard formats. Two input files are required: a cell-specific MIM in the CellDesigner XML format [15] and a generic metabolic network in the SBML format [16, 17]. Note that the MIM must be in an SBGN-compliant process description format [18] and the metabolic components should share the exact same identifier in both the MIM and the metabolic network (e.g. BiGG IDs [19], HMDB ID [20], MetaCyc ID [21]). MetaLo displays both control and cell- and/or disease-specific FBA results along with ratios of cellular ATP produced through glycolytic or oxidative pathways. It generates a variety of output files that include:

– A CaSQ-generated CSV file including all regulatory components names, logic formulae and aliases from CellDesigner;
– A CaSQ-generated SBML file encoding the Boolean model in the standard SBML-qual format;
– A CaSQ-generated BNET file encoding the Boolean model for further use by Trappist;
– A Trappist-generated CSV file covering all computed trap-spaces of the regulatory model;
– A MetaLo-generated CSV file displaying control FBA results;
– A MetaLo-generated CSV file displaying cell- and/or disease-specific FBA results.

MetaLo relies on four external packages (i.e. CaSQ [9], CobraPy [11], Pandas, and Trappist [10]) and provides the following additional options to: (i) infer a Boolean model only from specific sub-parts downstream and/or upstream of specific components of the MIM, (ii) provide an additional CSV file including initial conditions to forcefully initialize the regulatory model, and (iii) display flux variability (FVA) results instead of FBA results to provide metabolic fluxes intervals rather than precise values.

## 4 Applications

To benchmark the performance and evaluate the results of MetaLo, its FVA results for RASFs and breast CAFs were compared to the FBA results of the original publications [8, 22]. FVAs cover the whole range of optimized fluxes, while FBA returns only one of the possible solutions. The comparison was made to ensure that the FBA results of the published use cases lie within the MetaLo computed FVA flux-range.

### 4.1 Rheumatoid arthritis synovial fibroblasts

To study the impact of gene regulation and cell signaling machineries upon the metabolism of RASFs, we used MetaLo to automatically couple the Boolean regulatory model inferred from the RA-map V2 [23] with MitoCore’s [24] generic reconstruction of human central metabolism. However, the RA-map V2 is a global map, gathering information from several cell types, tissues, and fluids such as RASFs, synovial tissue, synovial fluid, blood and serum components, peripheral blood mononuclear cells, chondrocytes, and macrophages. Thus, several of MetaLo’s options were leveraged. First, we inferred a Boolean model exclusively from RASF-specific pathways within the RA-map, focusing on a designated set of components for exporting a subnetwork in both upstream and downstream directions, rather than considering the global map. Second, we initialized the Boolean network inferred from the latter submap in a cell- and disease-specific context through fixing its initial conditions (see Figure 2B and the tutorial on https://pypi.org/project/metalo/). Finally, we displayed FVAs results instead of the default FBA for comparison purposes.

MetaLo’s FVA results in terms of flux intervals cover the reaction flux value indicated by the FBA results of the initial framework. Moreover, the ratios of ATP production through glycolysis are equal between both analyses (i.e. 0.8505).

### 4.2 Breast-cancer associated fibroblasts

To study breast CAFs gene regulation and cellular signaling machineries impact upon metabolic fluxes distribution, we used MetaLo to automatically couple the Boolean regulatory model inferred from the CAF-map V2 [22] with MitoCore’s [24] generic reconstruction of human central metabolism. MetaLo’s additional arguments were used to initialize the regulatory model in a cell- and disease-specific context through a set of breast CAF-specific initial conditions and perform FVAs.

MetaLo’s FVA results in terms of flux intervals cover the reaction flux value indicated by the FBA results of the initial framework. Moreover, the ratios of ATP production through glycolysis are equal between both analyses (i.e. 0.8505).

## 5 Discussion

To decipher cellular phenotypes, it is essential to study metabolic networks in relation to their dynamic interactions with upstream signaling cascades and gene regulation. In this context, we present MetaLo, an open-source Python package coupling Boolean models inferred from MIMs, with generic metabolic networks. Translation of Boolean trap-spaces into additional metabolic constraints allows to contextualize a metabolic network and assess the impact of regulatory behavior upon metabolic flux distribution, and its energy production function. MetaLo enables to leverage the advantages of MIMs (i.e. high quality and manually curated static knowledge bases of interactions and entities) and Boolean model analysis (i.e. identification of asynchronous trap-spaces providing a comprehensive analysis of asymptotic behavior) in the generation of contextualized metabolic models. MetaLo efficiently handles large-scale molecular and metabolic networks with hundreds of components and can be applied to a wide range of biological contexts. Besides the command line, MetaLo is accessible via a graphical interface, minimizing the usability barrier for scientists less computer-savvy.
